# Metal–Organic Framework Integrating Ionic Framework and Bimetallic Coupling Effect for Highly Efficient Oxygen Evolution Reaction

**DOI:** 10.1002/advs.202203712

**Published:** 2022-09-01

**Authors:** Shulin Li, Tienan Wang, Dai Tang, Yuting Yang, Yuyang Tian, Fengchao Cui, Jifeng Sun, Xiaofei Jing, David S. Sholl, Guangshan Zhu

**Affiliations:** ^1^ Key Laboratory of Polyoxometalate and Reticular Material Chemistry of Ministry of Education Northeast Normal University Changchun 130024 China; ^2^ School of Chemical and Biomolecular Engineering Georgia Institute of Technology Atlanta GA 30332 USA; ^3^ Oak Ridge National Laboratory Oak Ridge TN 37830 USA; ^4^ School of Chemical and Biomolecular Engineering Georgia Institute of Technology Atlanta GA 30332 USA

**Keywords:** bimetallic coupling effect, density functional theory, ionic MOFs, metal–organic frameworks (MOFs), oxygen evolution reaction

## Abstract

Metal–organic frameworks (MOFs) are recognized as promising electrocatalysts for the oxygen evolution reaction (OER) because of their permanent porosity and rich architectural diversity; however, ionic MOFs enabling fast ions exchange during OER are rarely explored. Here, an ionic MOF (Ni‐btz) constructed with an azolate ligand is selected, and continuous 3D bimetallic MOF (NiFe‐btz) films deriving from high‐degree intergrowth of microsized MOFs particles are fabricated. The as‐prepared NiFe‐btz/NF‐OH electrode exhibits excellent OER performance with a low overpotential of 239 mV at 10 mA cm^−2^ under alkaline condition. The OER charge transfer process and bimetallic coupling effect in ionic NiFe‐btz are probed by density functional theory calculations and confirmed via X‐ray photoelectron spectroscopy and in situ Raman measurements. The partial density of states of NiFe‐btz indicates that the main contribution for electron density around the Fermi level is from Cl ions clarifying the profitable impact of ionic MOF framework. This work systematically demonstrates the relationship of electronic structure and OER activity in ionic, bimetallic MOFs and expands the scope of 3D MOF films for efficient OER.

## Graphical abstract

Ionic metal–organic frameworks (MOFs) enabling fast ions exchange are promising oxygen evolution reaction (OER) electrocatalysts. Here, Ni‐btz constructed from a neutral azolate ligand is selected and Fe is introduced to build up bimetallic coupling effect. Continuous 3D NiFe‐btz films derived from high‐degree intergrowth of macrosized MOF particles are fabricated, which are utilized as highly efficient OER electrocatalysts.

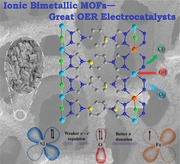

## 1 Introduction

Hydrogen is recognized as a clean energy carrier,^[^ ^1^, ^2^ ^]^ and developing electrocatalysts with high efficiency and low cost for water electrolysis is of great significance for hydrogen production.^[^ ^3^, ^4^ ^]^ However, the multistep proton‐coupled electron transfer process involved in the oxygen evolution reaction (OER) has caused slow and sluggish kinetics, creating a challenging issue for water splitting.^[^ ^5^, ^6^ ^]^ Precious metal catalysts including RuO_2_ and IrO_2_ are the current benchmark OER electrocatalysts, but their high cost and scarcity hinder their practical applications and motivate exploration of alternative earth‐abundant metal electrocatalysts such as NiFe‐based materials.^[^ ^7^, ^8^ ^]^

Metal–organic frameworks (MOFs), a family of crystalline porous materials constructed from metal ions (or clusters) and organic linkers, have attracted rising attentions for electrocatalysis.^[^ ^9^, ^10^, ^11^, ^12^, ^13^, ^14^ ^]^ Benefiting from their permanently porous skeletons, uniformly dispersed metal centers, and tunable lattice structures, MOFs can provide highly ordered channels for mass and electron transfer as well as abundant accessible active sites since MOFs can contain large numbers of redox‐active metal sites (Fe, Co, Ni, etc.).^[^ ^15^ ^]^ Unfortunately, as most pristine MOFs possess intrinsic poor conductivity, direct utilization of MOFs in powder state will encounter frustrated charge transfer during electrocatalytic reaction.^[^ ^16^, ^17^, ^18^, ^19^, ^20^, ^21^ ^]^ Great efforts on ultrathin MOFs nanosheets with enhanced OER activities have been carried out by using 2D MOFs or reducing the dimensionality of bulk MOFs,^[^ ^22^, ^23^ ^]^ however precise control of the synthesis of 2D MOF nanosheets with desired thickness along with high OER performance is still a great challenge.^[^ ^24^, ^25^ ^]^ These observations indicate the value of facile preparation of MOF films directly deriving from the intergrowth of MOF particles with strong OER activity.

Currently, various conductive substrates such as metal plates,^[^ ^26^ ^]^ metal foams,^[^ ^27^, ^28^ ^]^ graphene,^[^ ^29^ ^]^ and carbon fiber^[^ ^30^ ^]^ have been used to prepare pristine MOFs electrodes for OER. Among these options, nickel foam (NF) is a useful choice due to its porous texture and potential usage as metal source for MOFs’ preparation.^[^ ^31^ ^]^ Multiple NiFe‐based MOFs grown on NF have been reported as effective OER catalysts in which benzenedicarboxylic acid (BDC)^[^ ^28^, ^32^, ^33^ ^]^ and its derivatives^[^ ^31^, ^34^, ^35^ ^]^ are used as organic linkers, whereas ionic MOFs constructed from neutral ligands have been investigated far less.^[^ ^36^ ^]^ MOFs with ionic frameworks enable fast ions exchange under alkaline condition making them promising candidates for OER, and also suggesting that combining this concept with NF may be useful to obtain MOF films directly used in OER.

Here, in order to combine the advantages of bimetallic MOF and ionic framework for improved OER performance, we adopted an isoreticular framework to the Ni‐btz^[^ ^37^ ^]^ constructed from a neutral azolate ligand (1,4‐bis(4*H*‐1,2,4‐triazol‐4‐yl)benzene, btz) and built an ionic, bimetallic MOF (NiFe‐btz). We presented a semisacrificial template strategy for the in situ preparation of 3D ionic NiFe‐MOF films. A layer of uniform Ni(OH)_2_ is first prepared as a sacrificial Ni source which allows release of metal ions. Then continuous 3D MOF films are obtained via high‐degree intergrowth of MOF particles under neutral hydrothermal condition. The as‐prepared 3D NiFe‐btz/NF‐OH electrode can be directly applied as an OER catalyst in alkaline conditions and exhibits excellent OER performance with a low overpotential of 239 mV at 10 mA cm^−2^ and small Tafel slope of 44.3 mV dec^−1^ in 1 m KOH electrolyte. Meanwhile, the OER process in ionic NiFe‐btz and synergistic impact of ionic framework and bimetallic coupling effect are systematically investigated with density functional theory (DFT) and experimental detection of active intermediates by in situ Raman spectroscopy.

## 2 Results and Discussion

**Scheme 1:**
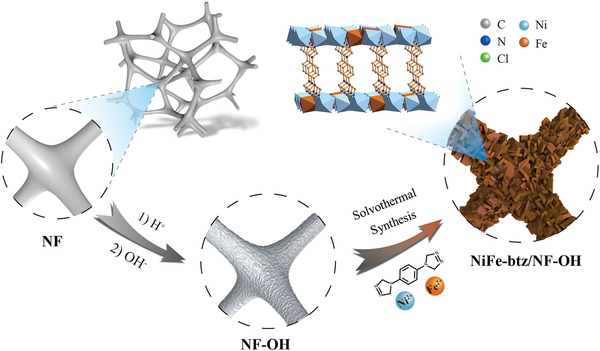
Illustration of the fabrication route of NiFe‐btz/NF‐OH electrode.

Ni‐btz is used as a potential electrochemical catalyst, benefitting from its abundant active sites, cationic framework and high surface area (Figure S1, Supporting Information). As illustrated in **Scheme** **1**, NF was treated with HCl followed by KOH solution to form a Ni(OH)_2_ layer on the surface of NF, which could serve as semisacrificial template for the preparation of continuous MOF films. Using a one‐step solvothermal method, NiFe‐btz/NF‐OH electrode derived from equimolar metal sources was successfully prepared in which iron ions were incorporated into MOF's skeleton to create bimetallic coupling effect. In addition, Ni‐btz/NF, NiFe‐btz/NF, Ni‐btz/NF‐OH, and Fe‐btz/NF‐OH electrodes were fabricated for comparison purpose, where M‐btz/NF and M‐btz/NF‐OH represent MOFs grow on untreated NF and treated NF with a Ni(OH)_2_ layer, respectively. Two more NiFe‐btz/NF‐OH from different ratios of metal sources were also prepared, which were denoted as NiFe‐btz‐1/NF‐OH (Ni:Fe = 9:1) and NiFe‐btz‐2/NF‐OH (Ni:Fe = 3:1).

**Figure 1:**
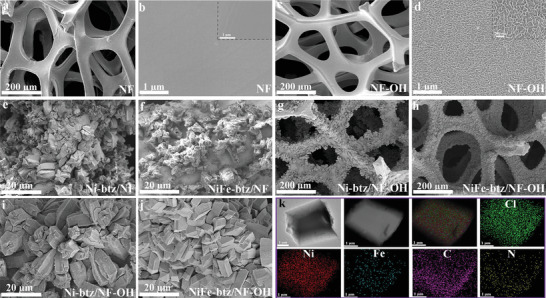
SEM images of a,b) NF, c,d) NF‐OH, e) Ni‐btz/NF, f) NiFe‐btz/NF, g,i) Ni‐btz/NF‐OH, and h,j) NiFe‐btz/NF‐OH. k) EDS element mapping of NiFe‐btz/NF‐OH.

Powder X‐ray diffraction (PXRD) was used to explore the crystalline structures of MOF films grown on the NF‐OH supports. As shown in Figure S2 (Supporting Information), all the PXRD patterns of M‐btz compounds are coincident with the simulated pattern of Ni‐btz (CCDC No. 1969913), which indicate that the as‐synthesized bulk MOFs are isostructural to Ni‐btz.^[^ ^38^ ^]^ In addition, all the diffraction peaks in M‐btz/NF‐OH are consistent with those of bulk MOFs, confirming the successful growth of MOFs on the surface of NF‐OH substrates. N_2_ adsorption–desorption isotherms of the activated MOF at 77 K show that the Brunauer–Emmett–Teller (BET) surface area of bulk NiFe‐btz is 1093 cm^2^ g^−1^ (Figure S3, Supporting Information), close to that of previously reported Ni‐btz treated with a similar method (1010 cm^2^ g^−1^). The morphologies of as‐prepared electrodes were investigated by scanning electron microscopy (SEM). Compared to the smooth surface of bare NF (**Figure** **1**a,b), Ni(OH)_2_ layer are uniformly formed over the surface of NF‐OH (Figure 1c,d) beneficial for the subsequent MOFs’ growth. X‐ray photoelectron spectroscopy (XPS) was performed to clarify the surface chemical compositions and their electronic states, and Ni‐OH and Ni^2+^ species are clearly detected in XPS spectra of Ni 2p and O 1s for NF‐OH substrate (Figure S4, Supporting Information). From the comparisons of MOF/NF and the corresponding MOF/NF‐OH SEM images (Figure 1e,i,f,j), MOF films grown on NF‐OH substrates are more closely packed on the substrate surfaces, profiting from the presence of Ni(OH)_2_ layers, which act as semisacrificial templates to provide Ni^2+^ ions and continuously arranged nucleation points. Compared to MOF grown on NF (Figure 1g), the intergrown degree of NiFe‐btz on NF‐OH is the best regardless of the Ni/Fe ratios (Figure 1h,j and Figure S5, Supporting Information). Element mapping of NiFe‐btz/NF‐OH was collected with energy‐dispersive X‐ray spectroscopy (EDS), showing that Ni, Fe, C, N, and Cl are uniformly distributed within NiFe‐btz films as desired (Figure 1k). Fe‐btz/NF‐OH fabricated without an additional Ni metal source exhibits less dense growth on NF‐OH substrates (Figure S6, Supporting Information). Ni/Fe ratios are calculated with the corresponding element percentages estimated from their EDS spectra (Figure S7 and Table S1, Supporting Information), and the results not only confirm the successful incorporation of Fe ions in M‐btz, but also prove the sacrificial template role of NF‐OH, as the Ni/Fe ratios of all MOF films are higher than the input ratios, even in the Fe‐btz film prepared with only Fe metal source in the solution. Unlike well‐investigated MOF nanosheets for OER, the NiFe‐btz/NF‐OH is a continuous 3D film composed of microsized MOF particles with high‐degree intergrowth, which may open up the exploration of 3D MOFs for OER.

**Figure 2:**
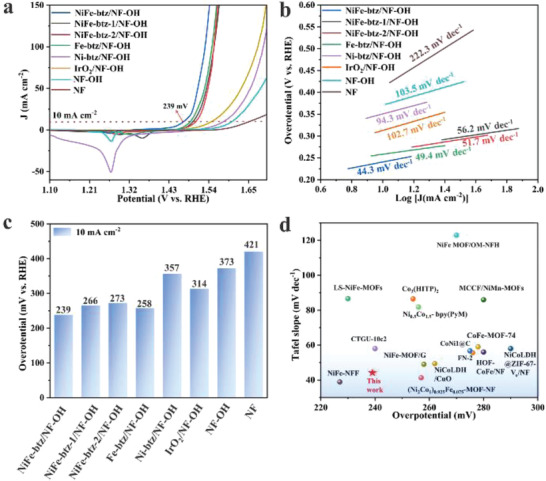
Electrocatalytic OER performance. a) LSV curves in the reverse sweep, b) corresponding Tafel slopes, and c) overpotentials at 10 mA cm^−2^ current densities of NiFe‐btz/NF‐OH, NiFe‐btz‐1/NF‐OH, NiFe‐btz‐2/NF‐OH, Fe‐btz/NF‐OH, Ni‐btz/NF‐OH, IrO_2_/NF‐OH, NF‐OH, and NF. d) Comparative overpotentials at 10 mA cm^−2^ of reported MOF‐based OER catalysts.

In order to verify their catalytic OER activities, the as‐prepared MOF electrodes with loading mass of 2.5–3.0 mg cm^−1^ were studied with a three‐electrode system in alkaline condition (1 m KOH). Linear sweep voltammetry (LSV) curves of MOFs grown on NF and NF‐OH supports were collected at a scan rate of 5 mV s^−1^, while a IrO_2_ catalyst on NF support was used for comparison. To exclude the possible interference from the anodic peak centered around 1.36 V caused by the oxidation of metal prior to water oxidation (Figure S8, Supporting Information), LSV curves collected in the reverse sweep were applied to extract the overpotential values at 10 mA cm^−2^ for all catalysts.^[^ ^39^ ^]^ NiFe‐btz/NF‐OH exhibits the best OER performance with the lowest overpotential of 239 mV at 10 mA cm^−2^ (**Figure** **2**a,c), outperforming those of Fe‐btz/NF‐OH (258 mV), Ni‐btz/NF‐OH (357 mV), NiFe‐btz/NF (253 mV), and other attempts (Figure S9, Supporting Information), also surpassing IrO_2_/NF‐OH (314 mV) and most reported MOF catalysts^[^ ^11^ ^]^ (Figure 2d and Table S2, Supporting Information). Tafel plots were extracted from the reverse scans to understand the OER kinetics. NiFe‐btz/NF‐OH presented the smallest Tafel slope of 44.3 mV dec^−1^, much lower than that of Ni‐btz/NF‐OH (94.3 mV dec^−1^), IrO_2_/NF‐OH (102.7 mV dec^−1^), demonstrating its faster reaction kinetics (Figure 2b). Benefiting from the synergistic effect between Ni and Fe active sites, favorable OER activity of NiFe‐btz/NF‐OH over Fe‐btz/NF‐OH (49.4 mV dec^−1^) is obtained, and the continuous growth of 3D MOFs on NF‐OH makes a contribution of faster OER kinetics than those grown loosely on NF (NiFe‐btz/NF, 53.5 mV dec^−1^). We also subsequently performed OER tests on the MOF films after pre‐exposure to 1 m KOH for 30 min, and both exhibited similar LSV curves during OER (Figure S10a, Supporting Information), except for the anodic peak centered around 1.26 V caused by the ion exchanges in ionic MOF. As shown in the Figure S10b (Supporting Information), Tafel plots indicated that NiFe‐btz/NF‐OH‐30 min presenting slightly increased reaction kinetics (from 44.3 to 41.3 mV dec^−1^), which was possibly due to the OH^–^ exchanged framework. The intrinsic activities of MOF electrodes were evaluated by comparing the electrochemical surface areas (ECSAs) calculated from double‐layer capacitance (*C*_dl_) via cyclic voltammetry (CV) scanned at different scan rates in the 0.2–0.26 V potential range without any redox processes (Figure S11, Supporting Information).^[^ ^40^ ^]^ NiFe‐btz/NF‐OH possesses higher *C*_dl_ value (6.02 mF cm^−2^) than Ni‐btz/NF‐OH (4.9 mF cm^−2^) and IrO_2_/NF‐OH (4.3 mF cm^−2^), indicating that it has larger active surface area and more exposed active sites for OER (Figure S12a, Supporting Information). Electrochemical impedance spectroscopy (EIS) was recorded to investigate the transport resistance of the electrodes. Nyquist plots obtained at 1.54 V (vs RHE) under OER condition (Figure S12b, Supporting Information) reveal a small solution ionic resistance (*R*_s_) of 2.3 ± 0.3 Ω for all samples, benefiting from the evenly distributed test systems and their excellent current‐collecting capability. Notably, NiFe‐btz/NF‐OH presents lower charge transfer resistance (*R*_ct_) of 1.6 Ω compared with Ni‐btz/NF‐OH (4 Ω) and IrO_2_/NF‐OH (3.5 Ω), which is much smaller than those of previously reported samples.^[^ ^13^ ^]^ A chronopotentiometric test of NiFe‐btz/NF‐OH for electrochemical stability revealed that the current density approached a steady state value of ≈9 mA cm^−2^ with a negligible decrease in a 12 h continuous test, where the decrease at the beginning could be ascribed to the formation of oxygen bubbles at the electrode surface that obstructed the approach path of electrolyte toward the active centers (Figure S13a, Supporting Information).^[^ ^28^ ^]^ The LSV curve after a 12 h test almost remains constant with the initial one indicating its long‐term catalytic performance (Figure S13b, Supporting Information). Furthermore, the compositions, structures and metal chemical states of NiFe‐btz/NF‐OH after *i*–*t* test were examined by high‐resolution transmission electron microscopy (HRTEM), TEM‐EDS, and XPS, respectively. The HRTEM pattern indicated the presence of NiOOH and NiFe‐btz in the NiFe‐btz/NF‐OH after *i*–*t* test (Figure S14a, Supporting Information). The TEM‐EDS test gave the element percentages in NiFe‐btz/NF‐OH after *i*–*t* test (Table S3, Supporting Information), which exhibited a similar Ni/Fe ratio to the results of SEM‐EDS, but an increased O content and decreased Cl content compared to those before OER test. In addition, the TEM elemental mapping images also evidenced the evenly distribution of each element and the decrease of Cl content (Figure S14b, Supporting Information). The XPS results (Figure S15a–c, Supporting Information) showed that the partial oxidation of Ni induced by the OER process, demonstrating the emergence of oxyhydroxide intermediates. The significant decrease of Cl content was consistent with the XPS data after OER (Figure S15d, Supporting Information). From the above data it can be concluded that only the surface region of the NiFe‐btz/NF‐OH was transformed into NiOOH active intermediate during OER. The NiFe‐btz/NF‐OH still retained its bimetallic composition and ionic framework, indicating its electrocatalytic stability. Overall, the high‐degree intergrowth of microsized NiFe‐btz particles leads to close‐packed 3D NiFe‐btz film on NF‐OH, and enable the decrease of OER activation energy thus enhancing its charge transfer ability and conductivity.

**Figure 3:**
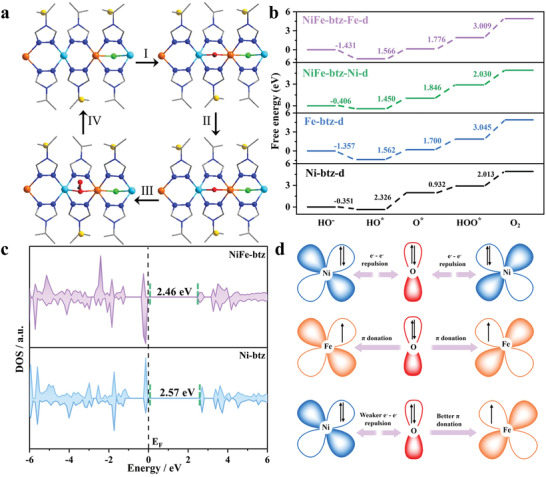
a) Elementary steps of the OER process in NiFe‐btz‐d, where two bridging Cl were removed to release the active metal sites. The light blue, orange, blue, green, yellow, and gray spheres represent Ni, Fe, N, bridging Cl, balance Cl and C atoms, respectively. b) The calculated free energy diagrams of the OER process in the absence of applied voltage for Ni‐btz‐d, Fe‐btz‐d, and NiFe‐btz‐d. c) Calculated DOS for pristine Ni‐btz and NiFe‐btz. d) Schematic representation of the electronic coupling between Ni and Fe in NiFe‐btz.

In order to better understand the synergistic effect of the ionic framework and bimetallic centers for enhanced OER, models of NiFe‐btz and Fe‐btz were built based on the crystal structure of Ni‐btz (Figure S16a, Supporting Information) and spin‐polarized DFT calculations were performed. In the proposed NiFe‐btz model, a quarter of the Ni atoms in the unit cell were replaced with Fe atoms to mimic our EDX results. The Ni atom adjacent to an Fe atom is labeled as Ni2, and the other two Ni atoms are labeled as Ni3 and Ni4, respectively. Different magnetic ordering patterns of each metal atom in these models were considered (Figure S16b, Supporting Information), and a ferromagnetic (FM) arrangement was adopted (Table S4, Supporting Information). As Cl element in M‐btz mostly disappeared after OER (Figure S17, Supporting Information), two active MOF models were considered to fully demonstrate the OER mechanism in this system. The first model is nondefect model with one bridging Cl between two metal sites removed, denoted as M‐btz‐n (Figure S18, Supporting Information), the removed Cl position is potential active site in this model. The second model is denoted as M‐btz‐d (Figure S19, Supporting Information), in which two bridging Cl on one metal are removed, therefore, one vacancy will be the active site and the other is considered as a defect. Also, NiFe‐btz‐Ni‐d (Figure S19c, Supporting Information) and NiFe‐btz‐Fe‐d (Figure S19d, Supporting Information) were investigated separately. Typically, there are four steps in OER mechanism under alkaline condition as shown in **Figure** **3**a: OH^−^ ions adsorption occurs followed by the formation of coupling between the *d* orbitals of metal atom and *p* orbitals of O atoms, which induces the formation of O‐containing intermediates such as HO^*^, O^*^, and HOO^*^ (step I–III, ^*^ represents the active sites), and then leads to production and desorption of O_2_ molecules (step IV). The Gibbs free energies as function of OER reaction pathway (see computational details in the Supporting Information) for each step in each MOF model were calculated. For M‐btz‐n model, the introduction of Fe produces smaller energy barriers (△*G*) in NiFe‐btz‐n (Figure S20, Supporting Information), but △*G*_4_ of the rate‐limiting step is still high. In M‐btz‐d models, we can see that NiFe‐btz‐Ni‐d possesses the lowest △*G* (2.030 eV) in the rate‐limiting step (Figure 3b), whereas Fe sites have the highest △*G* (3.009 eV for NiFe‐btz‐Fe‐d). Compared to Ni‐btz‐d, the rate‐limiting step for NiFe‐btz‐Ni‐d is altered from the second step (HO^*^ → O^*^) to the fourth one (HOO^*^ → O_2_) with Fe substitution suggesting the bimetallic coupling effect. These calculations indicate that defect model is more suitable for this OER system where defect Ni center adjacent to Fe is favorable active site in OER.^[^ ^9^ ^]^

Density of states (DOS) for Ni‐btz and NiFe‐btz were obtained from our DFT calculations. The computed bandgap is lowered from 2.57 eV (Ni‐btz) to 2.46 eV (NiFe‐btz) after Fe incorporation (Figure 3c). The partial density of states (PDOS) of these two MOFs (Figure S21, Supporting Information) shows that the PDOS of Cl 2p is the main contribution to the electron density around the Fermi level (E_F_), indicating the positive impact of the ionic framework for increased conductivity. The comparative PDOS for Ni centers in pristine Ni‐btz and NiFe‐btz (Figure S22a,b, Supporting Information) show that the energies above E_F_ of 3d *e_g_* and *t_2g_* orbitals of Ni atoms in NiFe‐btz both move toward E_F_, suggesting a more active character of NiFe‐btz. M‐btz models with one bridging Cl replaced by one OH^−^ ion, denoted as M‐btz‐O, were used to mimic the ongoing OER process in M‐btz, that is during OER one OH^–^ ion adsorbed in M‐btz accompanied by one bridging Cl expulsion. The PDOS of Ni 3d in M‐btz‐O were also tested (Figure S22c,d, Supporting Information), and the occupation of 3d *e_g_* and *t_2g_* states of Ni in these O‐containing models become lower in NiFe‐btz than in Ni‐btz, indicating potential electron transfer from Ni to Fe atoms during OER.^[^ ^41^ ^]^ The lower occupation of the unfilled 3d states of Ni in NiFe‐btz‐O compared to Ni‐btz‐O also suggests that Ni atoms in NiFe‐btz are more active in interacting with OER O‐containing intermediates, this is also verified in other reported Ni‐MOFs.^[^ ^33^ ^]^ The charge transfer process is consistent with the observed electronic structure (Figure 3d). After O‐containing intermediates are adsorbed around the metal sites, *e^−^ – e^−^* repulsion is the primary interaction between O atoms and Ni^2+^, as the *π*‐symmetry (*t_2g_*) *d*‐orbitals of Ni^2+^ are fully occupied. In contrast, the valence electronic configuration of Fe^3+^ is 3d_5_ with high‐spin state, unpaired electrons in its *π*‐symmetry (*t_2g_*) *d*‐orbitals would interact with O atoms via *π*‐donation. After the coupling between Ni^2+^ and Fe^3+^, *π*‐donation via Fe—O is strengthened by *e^–^ – e^–^* repulsion of Ni‐O, promoting the partial charge transfer from Ni^2+^ to Fe^3+^.

**Figure 4:**
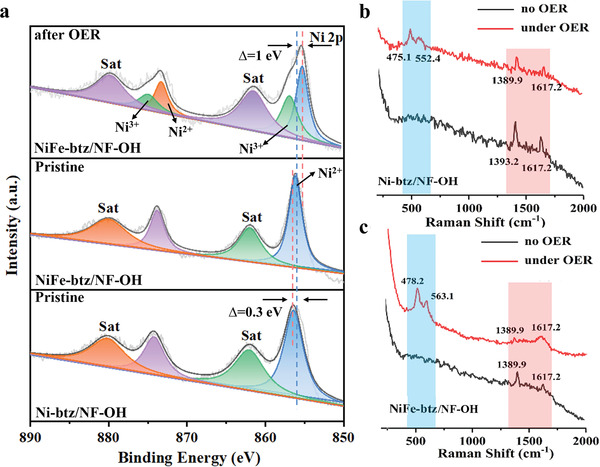
a) High‐resolution XPS spectra of Ni 2p in pristine Ni‐btz/NF‐OH, pristine NiFe‐btz/NF‐OH, and NiFe‐btz/NF‐OH after OER test. In situ Raman spectra of b) Ni‐btz/NF‐OH and c) NiFe‐btz/NF‐OH recorded before OER (black curves) and during OER test (red curves).

To further understand the bimetallic coupling, the survey XPS spectra of pristine NiFe‐btz/NF‐OH and NiFe‐btz/NF‐OH after OER test were collected (Figure S23, Supporting Information), which verify the coexistence of Ni, Fe, O, N, and Cl elements. Compared to the XPS Ni 2p spectra in Ni‐btz/NF‐OH, the peak of Ni^2+^ 2p_3/2_ shifted to higher binding energy in NiFe‐btz/NF‐OH (**Figure** **4**a) suggesting the partial electron transfer between Ni and Fe, consistent with the formation of bimetallic MOF structure and our PDOS results.^[^ ^10^, ^33^, ^42^ ^]^ It is noted that Fe^3+^ species is present in NiFe‐btz/NF‐OH due to the surface oxidation by exposure to ambient atmosphere (Figure S24, Supporting Information). After an OER test, the Ni 2p spectrum exhibits a down shift from 856.4 to 855.4 eV as well as the emergence of Ni^3+^ species at 856.9 eV (Figure 4a), the Fe 2p^3/2^ peak shifts higher by about 1 eV, the ratio of Fe^3+^/Fe^2+^ increases about 1.36 times (Figure S24, Supporting Information). In addition, the intensity of the N 1s peak is weakened and downshifted (Figure S25, Supporting Information). The above observations confirm the electronic redistribution during the electrochemical OER process and the formation of MOOH active intermediates.^[^ ^22^ ^]^ In situ Raman spectroscopy was employed to further explore the reaction intermediates during OER tests. In the black curves in Figure 4b,c, two pronounced peaks around 1389 and 1617 cm^−1^ are attributed to C=N and C=C bonds in the organic ligand of MOFs, respectively. As the overpotential increasing, the Raman spectra change significantly, with new peaks appearing in the range of 475–564 cm^−1^ which are associated with MOOH active intermediates.^[^ ^43^, ^44^ ^]^

## 3 Conclusion

In summary, ionic NiFe‐btz films were prepared in a straightforward way using a semisacrificial template strategy and directly applied as OER electrocatalysts. The acid‐base treated NF allows homogeneous release of metal ions and continuous growth of 3D MOF films on substrates. The as‐prepared NiFe‐btz/NF‐OH exhibits excellent OER performance with a low overpotential of 239 mV at 10 mA cm^−2^ and small Tafel slope of 44.3 mV dec^–1^ in 1 m KOH electrolyte. The integrated effects including nature of Ni‐btz ionic framework, introduction of Fe species along with close‐packed MOF films all appear to contribute to the performance of this catalyst. DFT calculations enable us understand the OER mechanism and charge transfer process in these ionic MOFs, providing additional evidence for the benefits of ionic framework and Ni—Fe coupling effects for enhanced OER activity. The changes of electronic states and formation of reaction intermediates during OER tests were confirmed by XPS analyses together with in situ Raman techniques. The approach we have demonstrated for preparation of continuous 3D MOF films and utilization of ionic MOFs will be useful for continued development of MOFs as electrocatalysts for OER.

## 4 Experimental Section

### Materials

1,4‐Bis(4*H*‐1,2,4‐triazol‐4‐yl)benzene (btz) (Nanjing Mole Medical Science and Technology Co. Ltd.), nickel foam (Jilin Tianxuan Economic and Trade Co. Ltd.), NiCl_2_·6H_2_O (Sigma‐Aldrich, 99%), and anhydrous FeCl_2_ (Energy‐Chemical, 99%) were used as received without further purification. Dimethyl sulfoxide (DMSO) and ethanol (EtOH) were purchased from the Beijing Chemical Works. All other reagents and solvents were purchased from local commercial suppliers.

### Preparation of M‐btz/NF Electrodes

The NF cleaned by EtOH and deionized water was treated with HCl solution (1.0 m), and then washed with EtOH and deionized water under ultrasonication. M‐btz/NF denotes M‐btz directly grows on the pretreated NF supports and includes Ni‐btz/NF and NiFe‐btz/NF. In a typical process, 0.2 mmol of btz was dissolved in 10 mL DMSO and 20 mL deionized water in beaker A, and 0.5 mmol of NiCl_2_·6H_2_O was dissolved with 30 mL deionized water in beaker B. After they dissolved completely, the solution in beaker A was transferred to beaker B, and two pieces of NF with the size of 1.0 × 0.8 cm^2^ were laid flat in beaker B. After heated at 120 °C for 12 h then cooled to room temperature, the obtained Ni‐btz/NF were washed with DMSO and EtOH thoroughly and dried at 120 °C. The preparation process of NiFe‐btz/NF is similar to the above procedure, except for the metal sources are NiCl_2_·6H_2_O (0.25 mmol) and FeCl_2_ (0.25 mmol).

### Preparation of M‐btz/NF‐OH Electrodes

NF‐OH was prepared by treating the cleaned NF with HCl solution (1.0 m) and KOH (2.0 m) in sequence. M‐btz/NF‐OH denotes M‐btz grown on NF‐OH supports and includes Ni‐btz/NF‐OH, NiFe‐btz/NF‐OH, NiFe‐btz‐1/NF‐OH, NiFe‐btz‐2/NF‐OH, and Fe‐btz/NF‐OH. The fabrication process of M‐btz/NF‐OH is similar to that of M‐btz/NF, and the total amounts of metal sources are all 0.5 mmol. The amounts of NiCl_2_·6H_2_O and FeCl_2_ are 0.45 and 0.05 mmol for NiFe‐btz‐1/NF‐OH, and 0.375 and 0.125 mmol for NiFe‐btz‐2/NF‐OH, respectively.

### Preparation of IrO_2_/NF and IrO_2_/NF‐OH Electrodes

5 mg IrO_2_ was dispersed in the mixture of 0.98 mL EtOH and 0.02 mL Nafion solution (5 wt%) to prepare IrO_2_ suspension. 0.1 mL of the suspension was dropped on the pretreated NF and NF‐OH supports with size of 1.0 × 0.8 cm^2^ and then dried at room temperature.

### Physical Characterization

PXRD measurements were conducted using Rigaku SmartLab X‐ray diffractometer with Cu‐K*α* radiation (40 kV, 30 mA, *λ* = 1.5418 Å) at a scanning step of 0.01°. N_2_ adsorption–desorption experiments were conducted at 77 K using a Quantachrome Autosorb‐iQ2 analyzer. The samples were exchanged with ethanol and then activated under vacuum at 80 °C for 4 h before sorption measurements. SEM was performed on FEI NovaNanoSEM450 and energy spectrum was tested with an OXFORD INSTRUMENTS X‐MAS. TEM was recorded with JEOL 2100F. XPS measurements were performed with a Thermo ESCALAB 250. Raman spectra were recorded with a homemade Raman spectrometer (SpectraPro HRS‐500 equipped with a PyLoN:100 CCD, Princeton Instruments) with a laser excitation wavelength of 633 nm and an acquisition time of 300 s.

### Electrochemical Characterization

All the electrochemical measurements were performed in a standard three‐electrode cell at room temperature using an electrochemical workstation (CH Instruments, CHI760E) in which the as‐synthesized electrodes were used as the working electrodes (geometric area: 0.8 cm^2^), a platinum plate (1 × 1 cm^2^), and Ag/AgCl (3.5 m KCl) were used as counter electrode and reference electrode, respectively. The electrolyte was 1.0 m KOH bubbled with N_2_ for 30 min prior to electrochemical measurements. All the measured potentials were converted to potentials referring to RHE according to the following equation: *E*_RHE_ = *E*_Ag/AgCl_ + 0.059 × pH + 0.205 V. LSV curves were recorded at a sweep rate of 5 mV s^−1^ and corrected with 95% *iR*‐compensation. EIS was carried out at 1.54 or 1.65 V versus RHE over the frequency ranging from 10^5^ to 0.1 Hz with the AC amplitude of 5 mV.

## Conflict of Interest

The authors declare no conflict of interest.

## Supporting information

Supporting information

## Data Availability

The data that support the findings of this study are available from the corresponding author upon reasonable request.
