# Supplementary material for: Metal–Organic Framework Integrating Ionic Framework and Bimetallic Coupling Effect for Highly Efficient Oxygen Evolution Reaction
Source: Adv Sci (Weinh). 2022 Sep 1;9(30):2203712. doi: 10.1002/advs.202203712 (PMC9596837; doi:10.1002/advs.202203712)
Supplement: Supplementary file 1 — Supporting information [file ADVS-9-2203712-s001.pdf]

ADVANCED SCIENCE
Open Access

Supporting Information

for *Adv. Sci.*, DOI 10.1002/advs.202203712

Metal–Organic Framework Integrating Ionic Framework and Bimetallic Coupling Effect for Highly Efficient Oxygen Evolution Reaction

Shulin Li, Tienan Wang, Dai Tang, Yuting Yang, Yuyang Tian, Fengchao Cui, Jifeng Sun, Xiaofei Jing, David S. Sholl* and Guangshan Zhu**

Supporting Information

Metal-Organic Framework Integrating Ionic Framework and Bimetallic Coupling Effect for Highly Efficient Oxygen Evolution Reaction

Shulin Li, Tienan Wang, Dai Tang, Yuting Yang, Yuyang Tian, Fengchao Cui, Jifeng Sun, Xiaofei Jing, David S. Sholl,* and Guangshan Zhu**

COMPUTATIONAL SIMULATIONS

We calculated the Gibbs free energy as function of reaction pathway of oxygen evolution reaction (OER) with spin-polarized DFT calculations using the Vienna ab initio Simulation Package (VASP5.4.4).[1] The approach of estimating free energy for OER by DFT is based on the method proposed by Norskov et al..[2-3] First, we benchmarked the effect of magnetic ordering of metal atoms on the system energy. There are four metal atoms in a unit cell of each MOF, with two of them forming a quasi 1-D chain and the other two atoms belonging to another parallel chain as shown in Figure S16a. For monometallic MOFs, Ni-btz and Fe-btz, we considered four types of magnetic ordering pattern in the crystallographic unit cell, one ferromagnetic (FM) arrangement and three anti-ferromagnetic (AFM) arrangements (Figure S16b). For bimetallic MOFs NiFe-btz, only one Fe atom was included in the unit cell because EDS results estimated the atomic ratio of Ni and Fe for NiFe-btz was about 4:1 (Table S1), so that the total possible FM and AFM arrangements of NiFe-btz were eight (Figure S16b). The initial magnetic moment of Ni and Fe was considered to be 3 μB. The Kohn-Sham wave functions were expanded in a plane wave basis set with a cutoff of 500 eV. The Brillouin zone was sampled by a 2×5×2 Gamma-centered *k*-point mesh. We allowed the relaxation of ionic positions and cell volume and shape until force change between ionic steps smaller than 0.03 eV/Å and energy change smaller than 10^{-4} eV per atom. The PAW method[4] and PBE potential[5] for the

exchange-correlation functional with D3 dispersion corrections[6] were used. The GGA+U approach[7-8] was used due to the consideration of the on-site Columbic repulsion for elements having *d* electrons, where the Hubbard *U* correction[9] was used for the localized *d* electrons with *U* values for Ni and Fe of 6.4 eV and 4.0 eV.[10-11] The cell parameters of a unit cell before structure relaxation are same for three MOFs, *a*=21.27 Å, *b*=7.074 Å, *c*=15.854 Å, *alpha*=90 deg, *beta*=90 deg, and *gamma*=90 deg. Table S4 lists the benchmark results. We found that the magnetic spin ordering of metal atoms had a negligible effect on the system energies of the MOFs we investigated, so we chose the FM spin ordering in all the following calculations.

We employed a 1×2×1 super cell of each MOF for Gibbs free energy calculations, in which Cl counterions were kept according to their crystal position. Two cases were considered to investigate the OER mechanism of each MOF. One is that the bridging Cl between two metals was removed (Figure S18), and the OER reactions will occur at the position of removed bridging Cl ion. The other case is that two bridging Cl ions were simultaneously removed to consider the defect effect for OER reaction (Figure S19), i.e., one removed Cl position acts as the OER reaction site and the other is as a defect site. For NiFe-btz, both Ni and Fe with the defect effect were considered, respectively, as shown in Figure S19c and S19d. Density of States (DOS) were calculated using pristine and one OH^- adsorbed NiFe-btz and Ni-btz models with 3×5×5 Gamma-centered *k*-point mesh.

Each system was geometry optimized using VASP by allowing the relaxation of ionic positions and cell volume and shape with FM spin ordering of metal atoms and a 2×2×2 Gamma-centered *k*-point mesh. The OER process includes 4 steps,

$* + OH^- \rightarrow HO^* + e^-$ (1)

$HO^* + OH^- \rightarrow O^* + H_2O + e^-$ (2)

$O^* + OH^- \rightarrow HOO^* + e^-$ (3)

$HOO^* + OH^- \rightarrow * + O_2 + H_2O + e^-$ (4)

where * represents the active sites. The initial position of the HO*/O*/HOO* groups was placed perpendicular to the *ab* plane and along the *c* direction. Separate calculations indicated that the HO*/O*/HOO* groups cannot attach to the metal sites if they were placed along the *a* direction and perpendicular to the *bc*-plane. The initial distance between O and each metal site was set to be 2.084 Å, the same as the Ni-O distance in bulk NiO. We calculated the binding energy of HO*, O* and HOO* for each system respectively. The Gibbs free energy ΔG of each OER step was calculated by

$$\Delta G = \Delta E + \Delta ZPE - T\Delta S + \Delta G_U \qquad (5)$$

where ΔE is the binding energy, ΔZPE is the zero-point energy correction, $T\Delta S$ is the entropy correction based on the vibrational entropy change at temperature T, and ΔG_U is energy correction due to the electrode potential *U*. The vibrational frequencies of the HO*/O*/HOO* groups were calculated by the configurations from the converged binding energy calculation. The $\Delta ZPE - T\Delta S$ term was then estimated using the vaspkit package at 298.15 K.[12] We did not compute the influence of pH since this would not differ among the set of materials we considered. The entropy corrections for adsorbates were also computed using vaspkit.[2, 12]

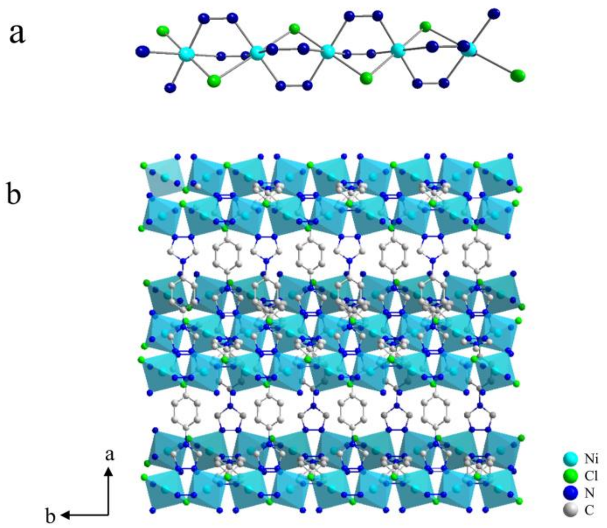

Figure S1. (a) The coordination environment of Ni-btz and (b) perspective view of Ni-btz along *c* axis.

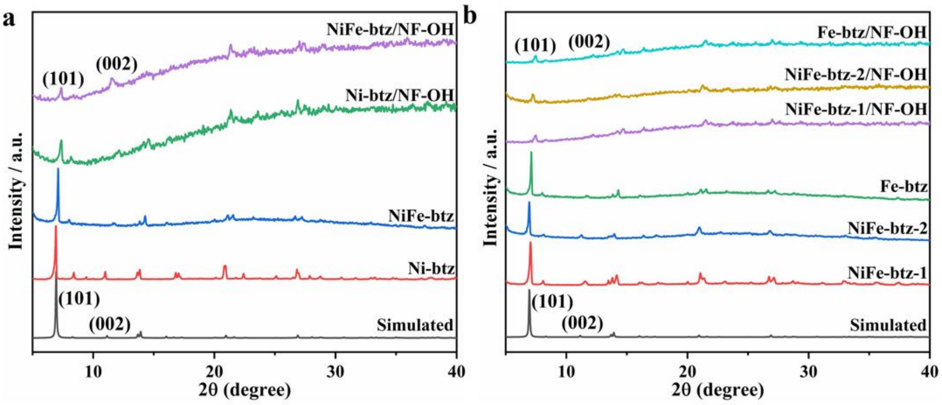

Figure S2. PXRD patterns of M-btz/NF-OH electrodes and the corresponding bulk MOFs synthesized under the same condition, including crystallographic simulation.

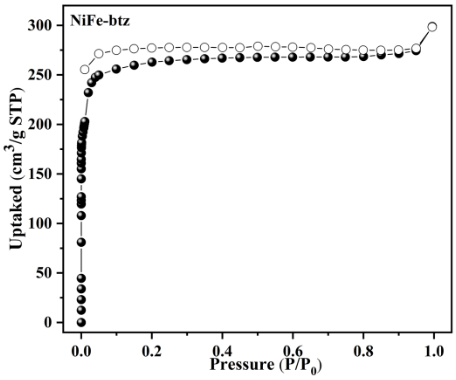

Figure S3. N_2 adsorption-desorption isotherms of activated NiFe-btz at 77 K.

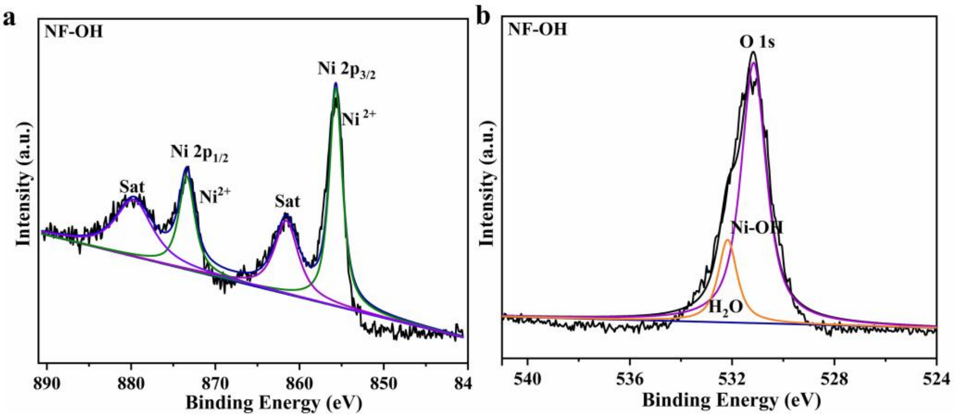

Figure S4. High-resolution XPS spectra of Ni 2p (a) and O 1s (b) in NF-OH substrate.

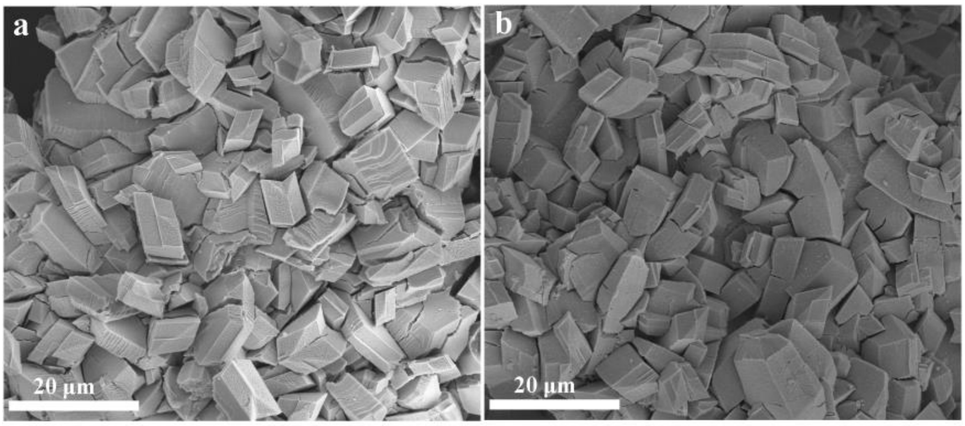

Figure S5. SEM images of NiFe-btz-1/NF-OH (a) and NiFe-btz-2/NF-OH (b).

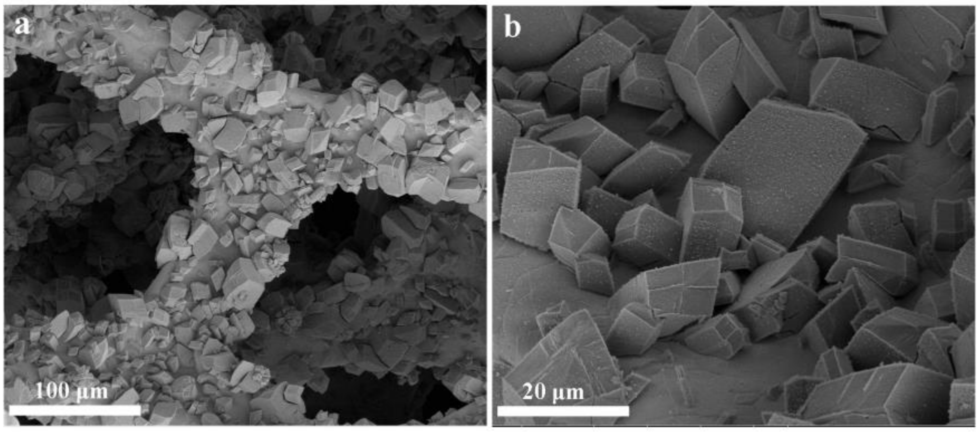

Figure S6. SEM images of Fe-btz/NF-OH.

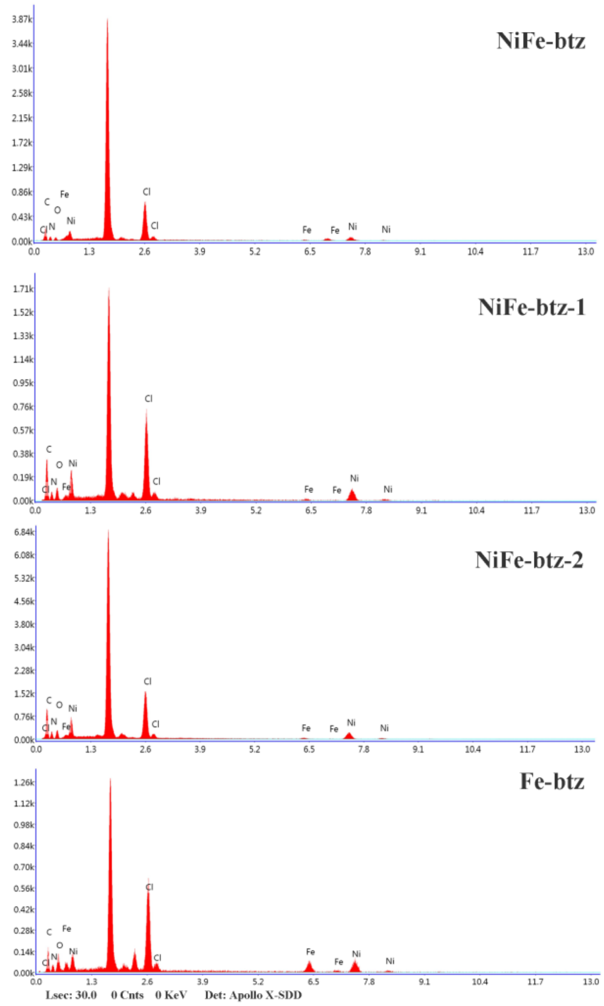

Figure S7. EDS spectra of NiFe-btz/NF-OH (a), NiFe-btz-1/NF-OH (b), NiFe-btz-2/NF-OH (c) and Fe-btz/NF-OH (d).

Table S1. The element percentages in MOFs grown on NF-OH estimated from their EDS spectra and the corresponding calculated Ni/Fe ratios.

**Element**	**NiFe-btz atomic %**	**NiFe-btz-1 atomic %**	**NiFe-btz-2 atomic %**	**Fe-btz atomic %**
C	49.34	51.46	48.99	44.09
N	29.92	26.37	29.33	24.55
O	13.72	15.17	15.58	21.14
Cl	4.54	4.69	3.96	6.05
Ni	1.99	2.15	1.93	2.57
Fe	0.49	0.15	0.22	1.59
Ni/Fe	4 : 1	14 : 1	9 : 1	1.6 : 1

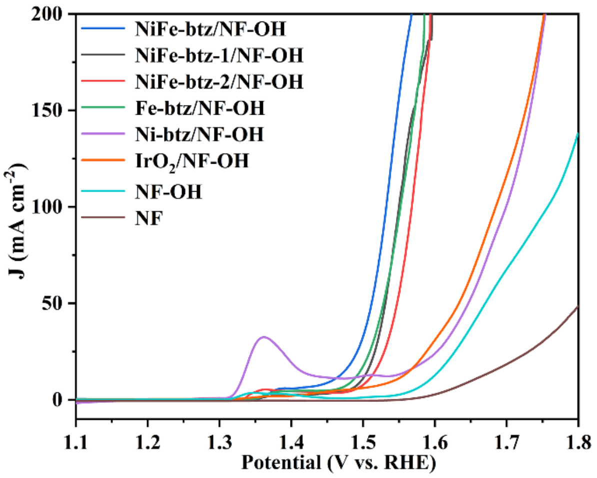

Figure S8. LSV curves of NiFe-btz/NF-OH, NiFe-btz-1/NF-OH, NiFe-btz-2/NF-OH, Fe-btz/NF-OH, Ni-btz/NF-OH, IrO_2/NF-OH, NF-OH and NF. The anodic peak centered around 1.36 V is caused by the oxidation of metal prior to water oxidation.

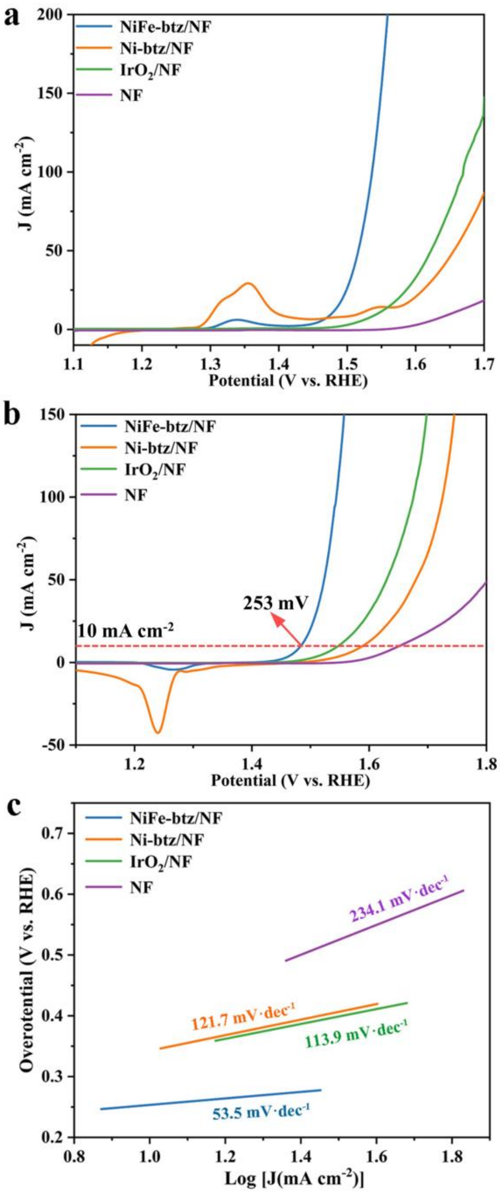

Figure S9. LSV curves (a), LSV curves in the reverse sweep (b) and corresponding Tafel slopes (c) of NiFe-btz/NF, Ni-btz/NF, IrO_2/NF and NF.

Table S2. OER performances for this work and reported MOF-based OER catalysts.

	**Overpotential (mV) at 10 mA cm^{-2}**	**Tafel slope (mV dec^{-1})**	**References**
NiFe-btz/NF-OH	239	44.3	This Work
CoFe-MOF-74	280	56	[13]
CTGU-10c2	240	58	[14]
LS-NiFe-MOFs	230	86.6	[15]
$(Ni_2Co_1)_{0.925}Fe_{0.075}$-MOF-NF	257	41.3	[16]
NiFe-NFF	227	38.9	[17]
NiFe MOF/OM-NFH	270	123	[18]
NiCoLDH/CuO	262	49.4	[19]
NiFe-MOF/G	258	49	[20]
$Co_3(HITP)_2$	254	86.5	[21]
MCCF/NiMn-MOFs	280	86	[22]
CoNi1@C	276	55.6	[23]
FN-2	275	56.7	[24]
Ni-Co LDH@ZIF-67-V_o/NF	290	58	[25]
HOF-$Co_{0.5}Fe_{0.5}$/NF	278	59	[26]

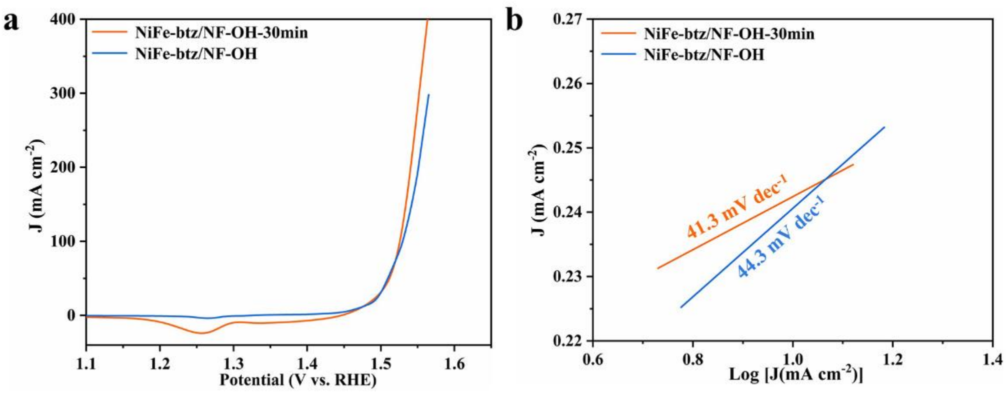

Figure S10. LSV curves in the reverse sweep (a) and corresponding Tafel slopes (b) of NiFe-btz/NF-OH and that immersed in 1 M KOH for 30 min before OER analysis.

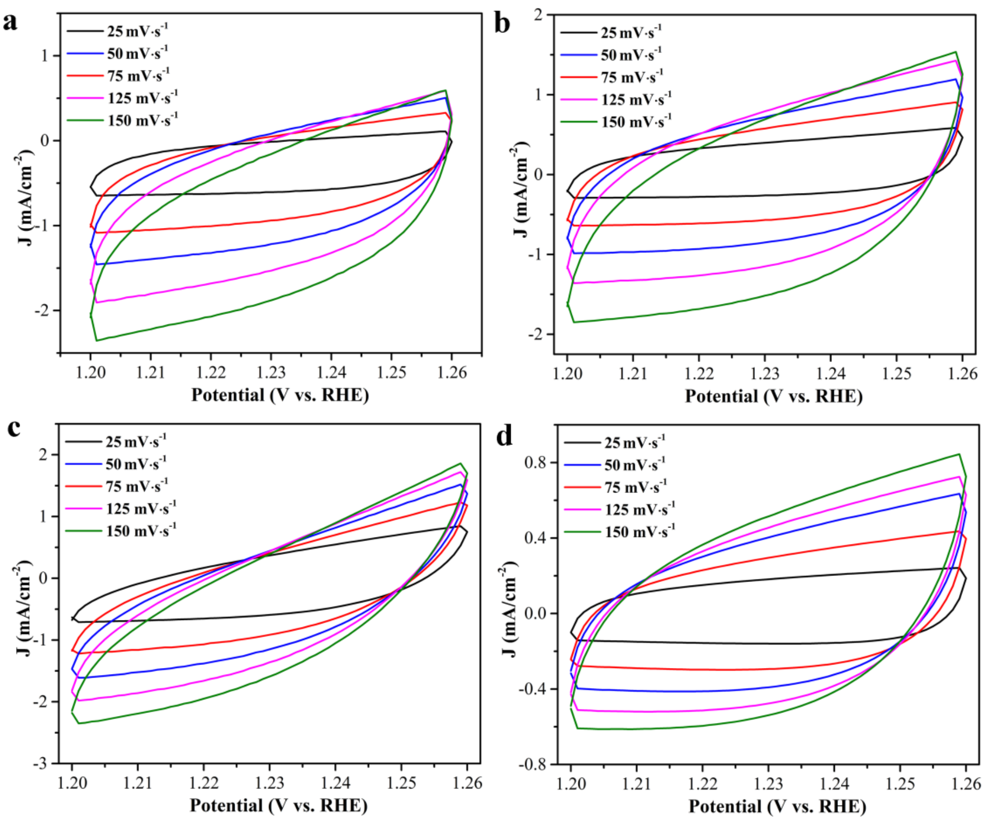

Figure S11. CV curves measured at different scan rates for (a) NF-OH, (b) Ni-btz/NF-OH, (c) NiFe-btz/NF-OH and (d) IrO_2/NF-OH.

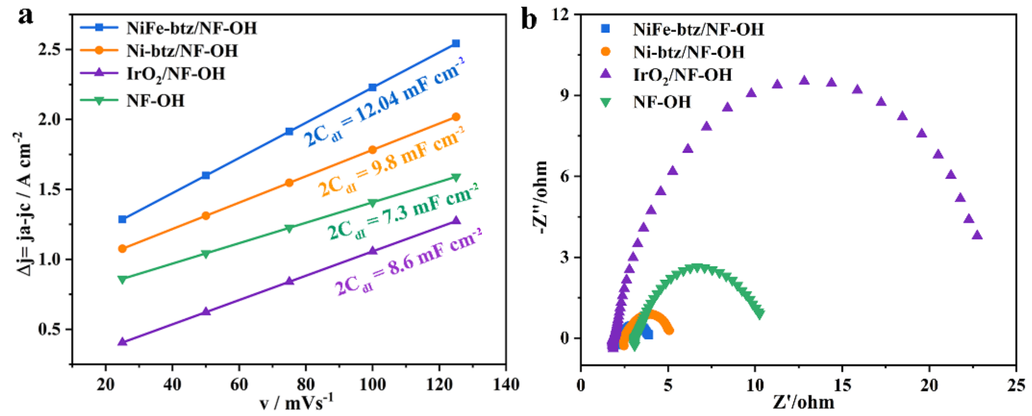

Figure S12. C_{dl} values (a) and Nyquist plots (b) of NiFe-btz/NF-OH, Ni-btz/NF-OH, IrO_2/NF-OH and NF-OH.

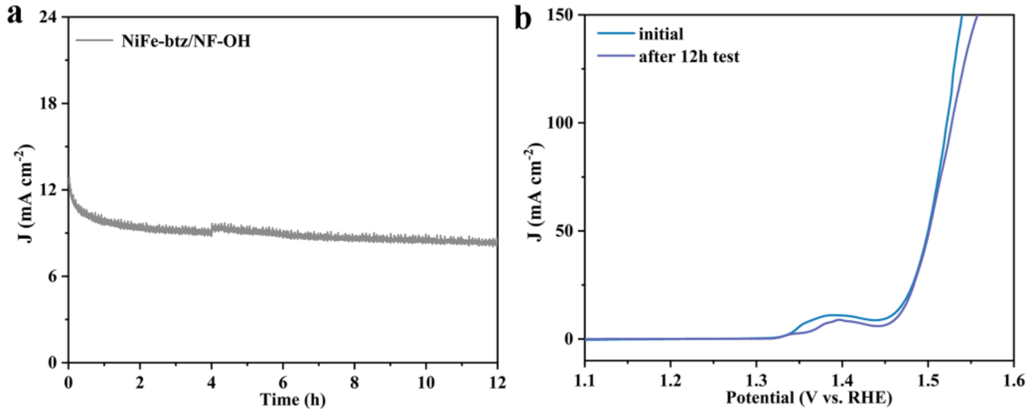

Figure S13. (a) Chronopotentiometry curve of NiFe-btz/NF-OH at 10 mA cm^{-2} in 1 M KOH. (b) LSV curves before and after 12 h stability test.

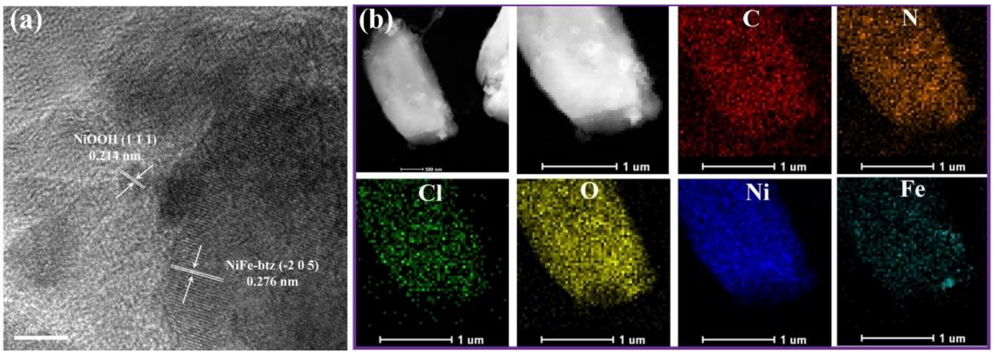

Figure S14 (a) HRTEM image and (b) TEM-element-mapping of NiFe-btz/NF-OH after *i-t* test.

Table S3. The element percentages in NiFe-btz/NF-OH after *i-t* 12 h test estimated from their TEM-EDS spectra

Element	Weight%	Atomic%	Uncert.%	k-Factor
C	54.18	70.51	0.43	3.94
N	3.71	4.14	0.27	3.826
Cl	0.09	0.04	0.01	1.063
O	19.79	19.33	0.18	1.974
Ni	18.29	4.87	0.14	1.511
Fe	3.92	1.09	0.06	1.403

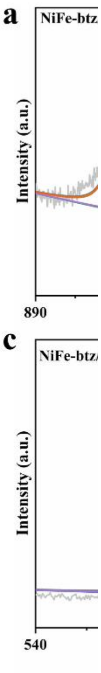

Figure S15 High-resolution XPS spectra of Ni 2p (a), Fe 2p (b), O 1s (c), Cl 2p (d) in NiFe-btz/NF-OH after *i-t* test.

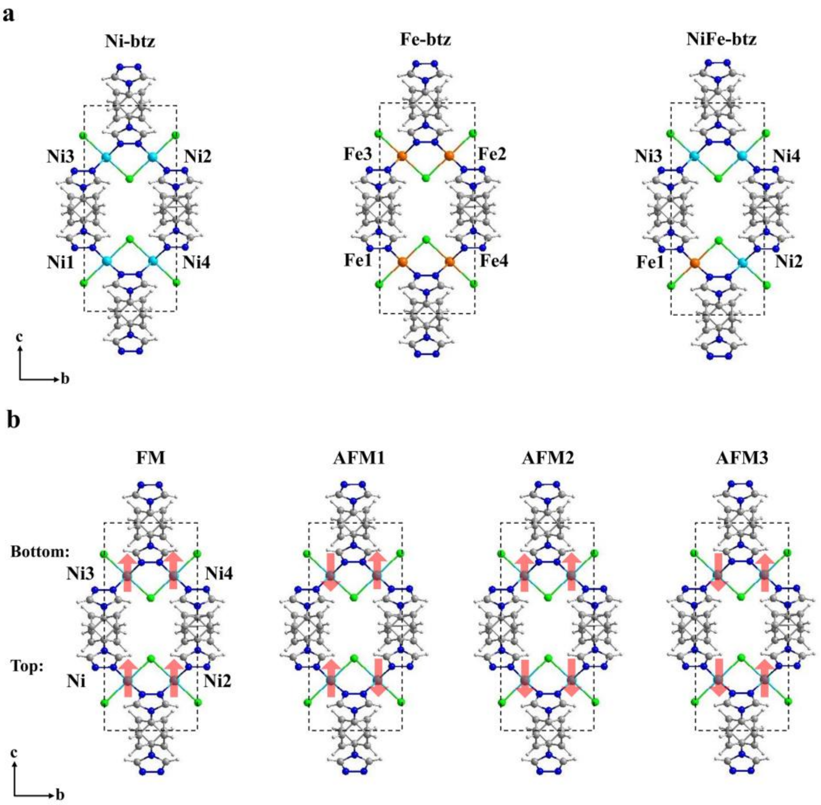

Figure S16. (a) Simulated models of Ni-btz, Fe-btz and NiFe-btz. The light blue, orange, blue, green, grey and white spheres represent Ni, Fe, N, Cl, C and H atoms, respectively. (b) Schematic magnetic ordering patterns of Ni atoms in the respective Ni-btz model. The up and down arrows show the relationship of spin polarization between Ni atoms. The balance Cl ions are omitted in all the structures.

Table S4. Different magnetic orderings, initial magnetic moment of metal atoms, total magnetic moment before/after simulation and energy of systems for Ni-btz, Fe-btz and NiFe-btz. The ordering of metal atoms of each MOF is consistent with that shown in Figure S16.

Ni-btz	Magnetic ordering	Initial magnetic moment of Ni (μB)	Total magnetic moment (μB)	Energy from DFT (eV)	Total magnetic moment from DFT (μB)
	No	-	-	-689.66568	-
	FM	3 3 3 3	12	-696.97899	8.4339
	AFM1	3 3 -3 -3	0	-697.00039	0
	AFM2	-3 3 3 -3	0	-697.00140	0
	AFM3	3 -3 3 -3	0	-697.00452	0
Fe-btz	Magnetic ordering	Initial magnetic	Total magnetic	Energy from DFT (eV)	Total magnetic

		moment (μB) of 4 Fe	moment (μB)		moment (μB) from DFT
	No	-	-	-706.12591	-
	FM	3 3 3 3	12	-710.46797	14.0616
	AFM1	3 3 -3 -3	0	-710.47074	0
	AFM2	-3 3 3 -3	0	-710.58953	0
	AFM3	3 -3 3 -3	0	-710.63559	0
NiFe-btz	Magnetic ordering	Initial magnetic moment (μB) of 3 Ni and 1 Fe	Total magnetic moment (μB)	Energy from simulation (eV)	Total magnetic moment (μB) from simulation
	no	-	-	-696.42745	-
	FM	3 3 3 3	12	-702.13063	5.4683
	AFM1	-3 -3 3 3	0	-702.11726	0.6510
	AFM2	-3 3 -3 3	0	-701.57416	1.4603
	AFM3	3 -3 -3 3	0	-702.01315	-2.1146
	AFM4	-3 3 3 3	6	-702.09094	2.5089
	AFM5	3 -3 3 3	6	-702.13257	-3.8353
	AFM6	3 3 -3 3	6	-702.11198	0.3850
	AFM7	-3 -3 -3 3	-6	-702.13254	-6.0737

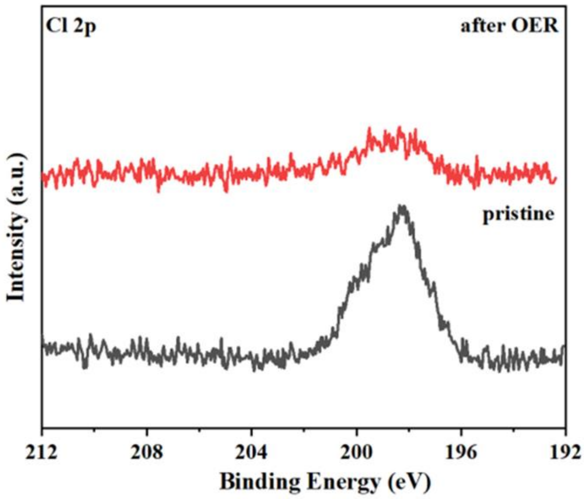

Figure S17. High-resolution XPS spectra of Cl 2p in pristine NiFe-btz/NF-OH and NiFe-btz/NF-OH after OER test.

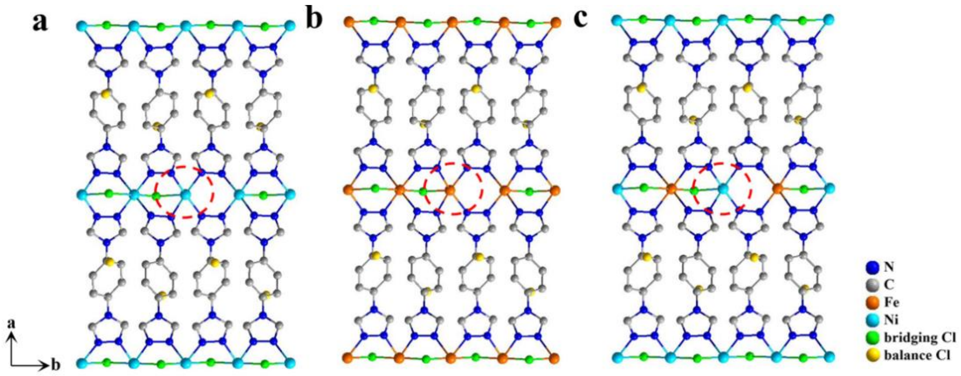

Figure S18. Representative 1×2×1 super cell of Ni-btz-n (a), Fe-btz-n (b) and NiFe-btz-n (c). The metal sites with one bridging Cl removed are circled with red dashed lines.

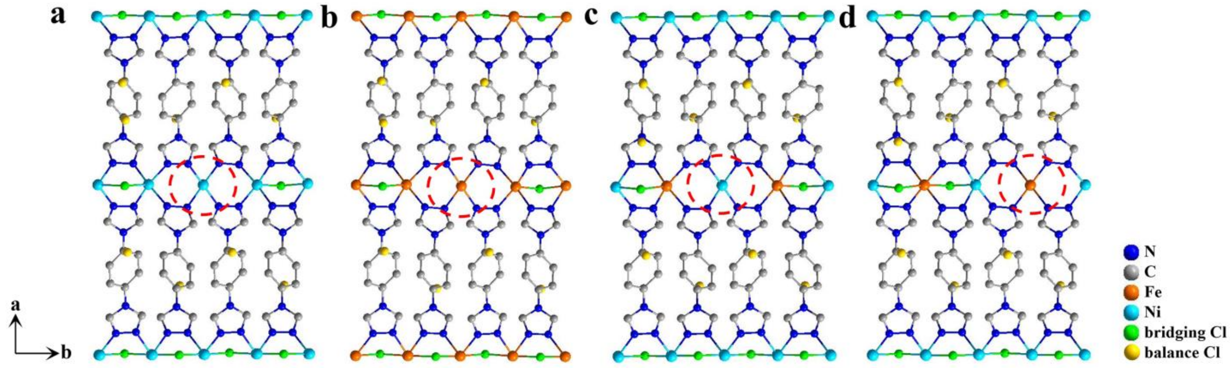

Figure S19. Representative 1×2×1 super cell of Ni-btz-d (a), Fe-btz-d (b) and NiFe-btz-d (c, d). The metal sites with two bridging Cl removed are circled with red dashed lines. In NiFe-btz-d, two active metal sites including NiFe-btz-Ni-d (c) and NiFe-btz-Fe-d (d) are considered separately.

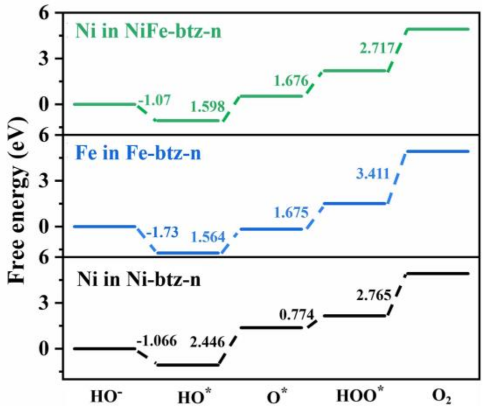

Figure S20. The calculated free energy diagrams at zero applied voltage of the OER process for active metal sites in Ni-btz-n, Fe-btz-n and NiFe-btz-n.

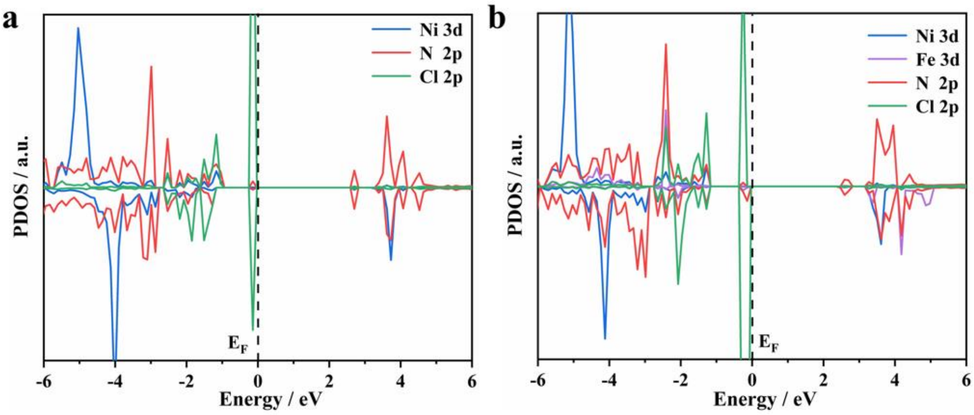

Figure S21. The calculated PDOS for Ni-btz (a) and NiFe-btz (b).

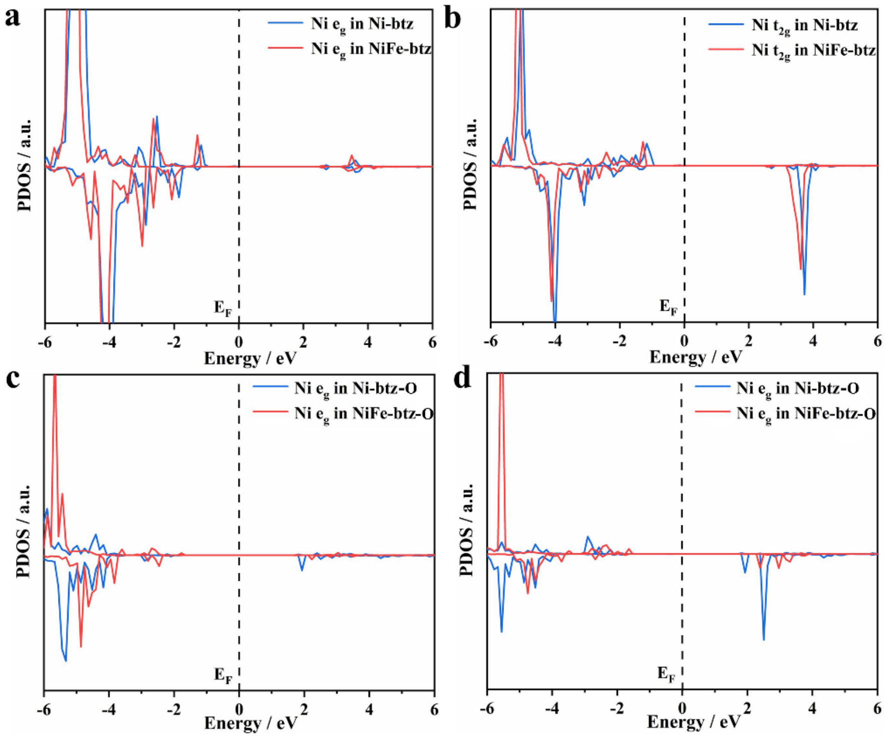

Figure S22. The calculated PDOS for Ni 3d e_g state (a) and Ni 3d t_{2g} state (b) in pristine M-btz models, and the calculated PDOS for Ni 3d e_g state (c) and Ni 3d t_{2g} state (d) in M-btz-O models with one bridging Cl replaced by one OH^- ion.

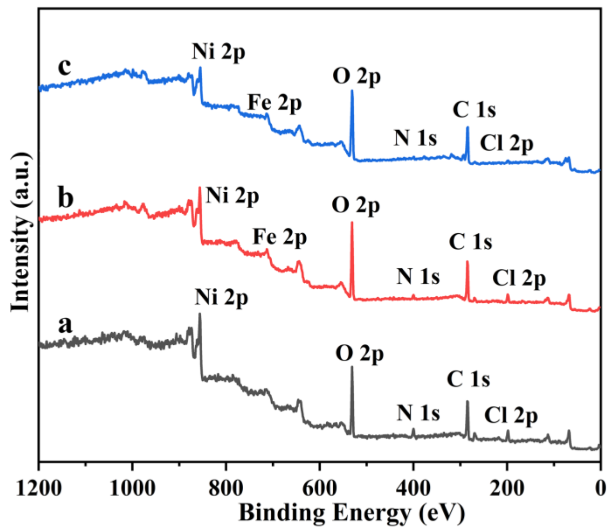

Figure S23. XPS survey spectra of pristine Ni-btz/NF-OH (a), pristine NiFe-btz/NF-OH (b) and NiFe-btz/NF-OH after OER test (c).

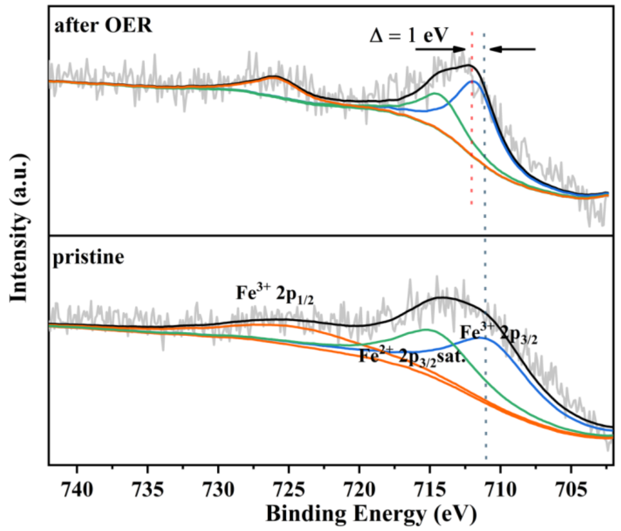

Figure S24. High-resolution XPS spectra of Fe 2p in pristine NiFe-btz/NF-OH and NiFe-btz/NF-OH after OER test.

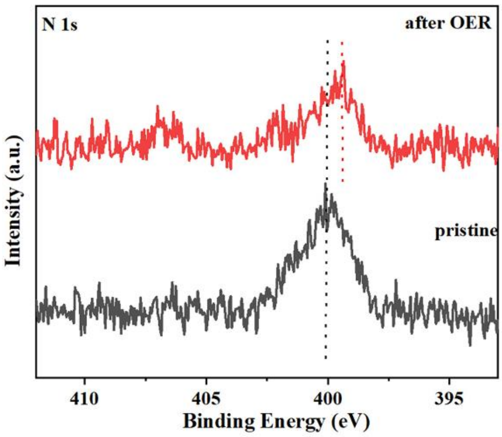

Figure S25. High-resolution XPS spectra of N 1s in pristine NiFe-btz/NF-OH and NiFe-btz/NF-OH after OER test.

Reference

[1] G. Kresse, J. Furthmuller, *Physical Review B* **1996,** *54*, 11169.

[2] J. K. Norskov, J. Rossmeisl, A. Logadottir, L. Lindqvist, J. R. Kitchin, T. Bligaard, *J. Phys. Chem. B* **2004,** *108*, 17886.

[3] A. Kulkarni, S. Siahrostami, A. Patel, J. K. Norskov, *Chem. Rev.* **2018,** *118*, 2302.

[4] P. E. Blochl, *Physical Review B* **1994,** *50*, 17953.

[5] J. P. Perdew, K. Burke, M. Ernzerhof, *Phys. Rev. Lett.* **1996,** *77*, 3865.

[6] S. Grimme, J. Antony, S. Ehrlich, H. Krieg, *J. Chem. Phys.* **2010,** *132*, 154104.

[7] S. Kummel, L. Kronik, *Rev Mod Phys* **2008,** *80*, 3.

[8] H. J. Kulik, M. Cococcioni, D. A. Scherlis, N. Marzari, *Phys. Rev. Lett.* **2006,** *97*, 103001.

[9] S. L. Dudarev, G. A. Botton, S. Y. Savrasov, C. J. Humphreys, A. P. Sutton, *Physical Review B* **1998,** *57*, 1505.

[10] L. Wang, T. Maxisch, G. Ceder, *Physical Review B* **2006,** *73*, 195107.

[11] G. W. Mann, K. Lee, M. Cococcioni, B. Smit, J. B. Neaton, *J. Chem. Phys.* **2016,** *144*, 174104.

[12] V. Wang, N. Xu, J. C. Liu, G. Tang, W. -T. Geng, *Comput. Phys. Commun*. **2021,** *267*, 108033.

[13] X. Zhao, B. Pattengale, D. Fan, Z. Zou, Y. Zhao, J. Du, J. Huang, and C. Xu, *ACS Energy Lett*. **2018**, 3, 2520.

[14] *Angew. Chem. Int. Ed*. **2019**, 58, 4227.

[15] Q. Ji, Y. Kong, C. Wang, H. Tan, H. Duan, W. Hu, G. Li, Y. Lu, N. Li, Y. Wang, J. Tian, Z. Qi, Z. Sun, F. Hu, W. Yan, *ACS Catal*. **2020**, 10, 5691.

[16] Q. Qian, Y. Li, Y. Liu, L. Yu, G. Zhang, *Adv. Mater*. **2019**, 31, 1901139.

[17] C. Cao, D.-D. Ma, Q. Xu, X.-T. Wu, Qi.-L. Zhu *Adv. Funct. Mater*. **2019**, 29,

1807418.
[18] X. Li, D.-D, Ma, C. Cao, R. Zou, Q. Xu, X.-Tao. Wu, Q.-Long. Zhu, *Small* **2019**, 15, 1902218.
[19] B. Chen, Z. Zhang, S. Kim, M. Baek, D. Kim, K.Yong, *Appl. Catal., B* **2019** 259 11801.
[20] Y. Wang, B. Liu, X. Shen, H. Arandiyan, T. Zhao, Y. Li, M. Garbrecht, Z. Su, L. Han, A. Tricoli, C. Zhao, *Adv. Energy Mater*. **2021**, 2003759.
[21] D.Xing, Y. Wang, P. Zhou, Y. Liu, Z. Wang, P. Wang, Z. Zheng, H. Cheng, Y. Dai, B. Huang, *Appl. Catal., B* **2020**, 278, 119295.
[22] W. Cheng, X. F. Lu, D. Luan, X. W. Lou, *Angew. Chem. Int. Ed*. **2020**, 59, 18234.
[23] X. Zhang, J. Luo, K. Wan, D. P, B. Sels, J. Song, L. Chen, T. Zhang, P. Tang, J. R. Morante, J. Arbiol, J. Fransaer, *J. Mater. Chem. A*. **2019**, 7, 1616.
[24] M. Liu, L. Kong, X. Wang, J. He, X.-H. Bu, *Small* **2019**, 15, 1903410.
[25] W. Chen, Y. Zhang, R. Huang, Y. Zhou, Y. Wu, Y. Hu, K. Ostrikov, *J. Mater. Chem. A*. **2019**, 7, 4950.
[26] F. Q. Liu, J. W. Liu, Z. Gao, L. Wang, X.-Z. Fu, L. X. Yang, Y. Tao, W. H. Yin, F. Luo, *Appl. Catal., B* **2019**, 258, 117973.
